# Snakebite epidemiology and health-seeking behavior in fringe communities of the Kakum Conservation Area in Ghana

**DOI:** 10.1371/journal.pntd.0013288

**Published:** 2026-10-30

**Authors:** Derek Anamaale Tuoyire, Linus Baatiema, Robert Peter Biney, Justus Precious Deikumah, John Koku Awoonor-Williams, Mawuli Kotope Gyakobo

**Affiliations:** 1 Department of Community Medicine, School of Medical Sciences, University of Cape Coast, Cape Coast, Ghana; 2 Ghana Health Service, Upper West Regional Health Directorate, Wa, Ghana; 3 Centre for Migration, Security and International Relations, Simon Diedong Dombo University of Business and Integrated Development Studies, Wa, Ghana; 4 Department of Pharmacotherapeutics and Pharmacy Practice, School of Pharmacy and Pharmaceutical Sciences, University of Cape Coast, Cape Coast, Ghana; 5 Department of Conservation Biology and Entomology, School of Biological Sciences, University of Cape Coast, Cape Coast, Ghana; 6 Faculty of Public Health, Ghana College of Physicians and Surgeons, Accra, Ghana; 7 Department of Internal Medicine and Therapeutics, School of Medical Sciences, University of Cape Coast, Cape Coast, Ghana; 8 Faculty of Family Medicine, Ghana College of Physicians and Surgeons, Accra, Ghana; Universidad de Costa Rica, COSTA RICA

## Abstract

**Background:**

Snakebite envenomation (SE) is a significant yet under reported public health challenge, especially in rural and forest-fringe communities in sub-Saharan Africa. Despite its recognition by the World Health Organization (WHO) as a Neglected Tropical Disease (NTD), epidemiological data on SE remain scarce in Ghana, particularly in communities fringing protected forest areas such as the Kakum Conservation Area (KCA).

**Objective:**

This study sought to examine snakebite epidemiology in the fringe communities of the Kakum Conservation Area (KCA) in the Central region of Ghana.

**Methods:**

A cross-sectional household survey was conducted between April and May 2024 in 18 communities fringing the KCA in Ghana’s Central Region. The data collection instrument was pretested in a non-study community (n = 30), with Cronbach’s alpha ranging from 0.72 to 0.86 for key scales, indicating acceptable internal consistency. A two-stage sampling approach was employed to enumerate a total of 1,445 households for snakebite victims. Descriptive statistics included frequencies, proportions, means, and standard deviations with 95% confidence intervals calculated using binomial approximation. Inferential statistics included chi-square tests for bivariate associations and multivariable logistic regression to calculate adjusted odds ratios (AOR) with 95% confidence intervals.

**Results:**

In this study, the reported lifetime occurrence of snakebites was 394/8,518 (4.63%, 95% CI: 4.18–5.07), compared with 164/8,518 (1.93%, 95% CI: 1.63–2.22) and 41/8,518 (0.48%, 95% CI: 0.33–0.63) in the preceding five years and one year, respectively. The mean age of victims was 44.69 (SD ± 18.64) years. Most victims were female (212/394, 53.81%), married (231/394, 58.63%) and engaged in farming (289/394, 73.35%). Most bites occurred in farms/forests (258/394, 65.48%) and affected the lower limbs (345/394, 87.56%). First aid was applied by 295/394 (74.87%) victims, predominantly involving tourniquets (103/295, 34.92%). Overall, 239/394 (60.66%) sought treatment, of whom 170/239 (71.13%) accessed formal healthcare facilities. Among treatment seekers, 124/239 (51.88%) travelled by motor vehicle, while 97/239 (40.59%) travelled on foot. Additionally, 92/239 (38.49%) waited at least one hour before seeking care, while 89/239 (37.24%) spent at least one hour travelling for care. Following snakebite, 373/394 (94.67%) victims recovered, 15/394 (3.81%) experienced disability and 6/394 (1.52%) died. Compared with victims aged under 20 years, those aged 30–39 years had higher adjusted odds of experiencing snakebite in farm/forest settings (AOR = 3.39, 95% CI: 1.07–10.67). Farming was also associated with increased odds of farm/forest snakebite incidents (AOR = 1.88, 95% CI: 1.04–3.40). Regarding facility-based care, victims aged 20–29 years (AOR = 0.20, 95% CI: 0.05–0.88) and ≥60 years (AOR = 0.11, 95% CI: 0.02–0.69) had lower adjusted odds of seeking care compared with those aged under 20 years.

**Conclusion:**

Snakebites constitute a major public health concern in communities fringing forested conservation areas, especially for people whose livelihood activities revolve around agricultural activities. Overall, the study underscores the need to establish and strengthen collaborative efforts among primary health authorities, forest conservation managers, and community leaders in the design and implementation of effective interventions to avert snakebite incidents and improve outcomes for victims.

## Author summary

Snakebite envenomation is a serious yet underreported public health issue particularly in rural forest-fringe communities in Africa. Despite being reclassified by the World Health Organization as a Neglected Tropical Disease, snakebite remains poorly documented in Ghana, especially in communities bordering protected forests where human-snake encounters are more likely.

We surveyed over 1,400 households in 18 communities surrounding the Kakum Conservation Area in southern Ghana. We found a lifetime snakebite prevalence of 4.63% (394 cases among a population of 8,518 people), with six deaths resulting. Most victims were adult farmers working on their farms or in nearby forests. Bites typically occurred on the limbs and were often followed by symptoms such as pain, swelling and bleeding. While over two-thirds of victims received some form of formal medical care, some continued to seek traditional remedies even after visiting a health facility.

Our findings suggest snakebite is a persistent health threat in rural forest-fringe communities and that health-seeking behavior after snakebite is shaped by a combination of factors including accessibility to healthcare. Therefore, strengthening rural health systems and improving community education at the local level are crucial steps to reduce the burden of snakebite in Ghana and similar settings.

## Background

Snakebite envenomation (SE) has increasingly become a public health concern, warranting its re-inclusion on the list of Neglected Tropical Diseases (NTDs) by the World Health Organization (WHO) in 2017 [1,2]. Although the actual number of snakebites and envenomation remain unknown, between 4.5 and 5.4 million people are estimated to experience snakebites annually across the world, accounting for about 400,000 disabilities with mortality ranging from 81,000–138,000 [3–5]. Given that human-snakebite encounter typically occur during field related agricultural activities where there are high grass or forests among other factors, snakebites disproportionately affect those residing in rural communities in low- and middle-income countries in Asia, Latin America and Africa [6,7]. In sub-Saharan Africa, snakebite envenomation afflicts an estimated 90,000–400,000 people annually, resulting in 3,500–32,000 deaths among victims [8–10].

Snakebite envenomation appears to be associated with livelihood activities given that victims are largely reported to be young and economically active, who are involved in subsistence farming, with bite sites often located on their extremities [11]. Given their underprivileged backgrounds, majority of victims are usually unable to seek prompt treatment in appropriate healthcare facilities, due in part to long distance and associated healthcare costs, poor knowledge and skepticism about the effectiveness of conventional modern medicine in treating snakebite envenomation [11,12]. As such, victims often seek help from traditional healers [12]. These healers offer remedies including use of tourniquets, incision at the site of bite, application of topical and emetic medicinal herbal concoctions or application of the black stone [12,13]. The remedies provided may not be effective and could compound the complications of snakebite envenomation. Thus, reinforcing a vicious cycle of poverty, livelihood activities, snakebite envenomation and ineffective health seeking behaviorss resulting in dire outcomes for victims. Survivors may suffer physical consequences such as life-long disabilities including amputations [14,15]. The psychological trauma, limitations on work and overall quality of life of snakebite victims cannot be overemphasized [15].

As in other regions across the globe, there is paucity of research and epidemiological data on the burden of snakebite envenomation in Ghana. Available studies vary in their reporting of the burden of snakebite envenoming. For instance, in northern Ghana, Musah et al. [11] estimated the prevalence of snakebite to be approximately 6%, with case fatality rate of 3% [11], while Yakubu et al. [16] reported about 86 envenomings, with an annual mortality estimated at 24/100,000 [16]. Another study by Punguyire et al [17] found a snakebite incidence of 92/100,000 in the Brong-Ahafo region. A more recent review based on data from the District Health Information and Management Systems (DHIMS) in the Oti region of Ghana between 2014 and 2018 reported a total of 2,973 cases of snakebite, with a 5-year average incidence of 24/100,000 [18].

While these studies provide some useful epidemiological insights into snakebite envenomation in Ghana, there is a dearth of similar studies in communities located within the fringes of forested areas, which typically serve as habitat for variety of distinct snake fauna and present unique human-snake interactions. Inhabitants of such communities are particularly more vulnerable to snakebite envenomation, as they tend to live in poor housing conditions within the frontiers of natural snake habitats, while engaged in subsistence agricultural activities and often several hours away from the nearest functional healthcare facility [19].

This study sought to examine snakebite epidemiology in the fringe communities of the Kakum Conservation Area (KCA) in the Central region of Ghana. Specifically, the study estimated the prevalence of snakebite, characterized snakebite victims, snake features, health seeking-behaviors, and examined associated factors. In this study, we use the term ‘fringe communities’ to refer to rural settlements located directly along the physical boundary of the KCA. These are forest-edge communities situated adjacent to protected forest habitat, rather than communities on the margins of mainstream society or in rural–urban transition zones. Their proximity to dense vegetation and wildlife corridors increases the likelihood of human–snake encounters.

The KCA is a protected area of moist semi-deciduous forest which forms part of the Upper Guinean Forest biodiversity hotspot of West Africa [20]. Cognizant of the WHO’s recognition of the lack of epidemiological awareness of snakebite envenomation and commitment to halving deaths and disabilities from snakebites by 2030 [6], this study would provide useful insights and contribute to the body of knowledge.

## Methods

### Ethics statement

Ethical approval, reference UCCIRB/EXT/2023/22 for the study was obtained from the University of Cape Coast Institutional Review Board. The approval was valid from 6^th^ October 2023–5^th^ October 2024. Prior to data collection, written formal informed consent was sought and obtained from all participants. For participants who were minors or incapacitated due to snakebite-related complications, written formal informed consent was obtained from a parent, guardian, or responsible adult household member. Participation in the study was entirely voluntary, and respondents were informed of their right to withdraw at any stage without a penalty. All data were anonymized to ensure confidentiality and were securely stored to prevent unauthorized access. Interviews were conducted in the participants’ preferred language (English, Fante/Twi) to facilitate comprehension and comfort. The study adhered to the ethical principles outlined in the Declaration of Helsinki.

### Study setting

The study was conducted in communities fringing the KCA, located within the Central region of Ghana. The KCA is located at 5.4167N and -1.3167W, covering an estimated 360 km^2^ comprising of two contiguous reserves – the Kakum National Park (210 km^2^) and the adjacent Assin Attandanso Resource Reserve (150 km^2^). This combined protected area forms part of the Upper Guinean Forest biodiversity hotspot of West Africa [20] and is characterized by three principal forest types: moist forest, edaphic forest, and boval forest [21,22]. The moist forests can be divided into two subgroups: *Lophira alata–Triplochiton scleroxylon* and *Celtis zenkeri–Triplochiton scleroxylon.* The edaphic forests are comprised of swamp and riverine forests. The population of the area is about 119,359 with about 64 fringe communities.

The forest-fringe communities surrounding the KCA were selected because they constitute one of the largest, most ecologically diverse rural populations living directly adjacent to a protected forest reserve in southern Ghana [19,21]. These communities experience frequent human–wildlife interactions, rely heavily on subsistence agriculture, and share socioeconomic characteristics common to forest-edge communities nationwide. While ecological and cultural variability exists across Ghana, the KCA fringe is widely recognized as representative of the broader forest-interface settlements where snakebite risk is elevated due to occupational exposure, dense vegetation, and proximity to wildlife habitat.

### Study population and sampling

The study targeted two categories of participants in the peripheral communities within the KCA forest enclave. The first consisted of snakebite victims, while the other category consisted of community members with experience of managing snakebite victims. A two-stage cross-sectional survey approach was employed in the selection of participants in the first phase of data collection involving snakebite victims. To ensure ecological and geographic representativeness, we adopted a probability approach. First, the 64 communities were stratified by geo-cardinal zones. From each zone, at least two communities were selected using simple random sampling. Sampled communities were selected to represent each geo-cardinal points (North, South, East, West, North-East, North-West, South-East and South-West) surrounding the forest using simple random sampling and Arc-GIS tool. A total number of 18 communities that collectively represented the full spatial distribution, ecological gradient, and demographic variability of the entire forest-fringe population (Fig 1) were selected from which 1,445 households were enumerated for the purpose of the current analyses.

**Fig 1:**
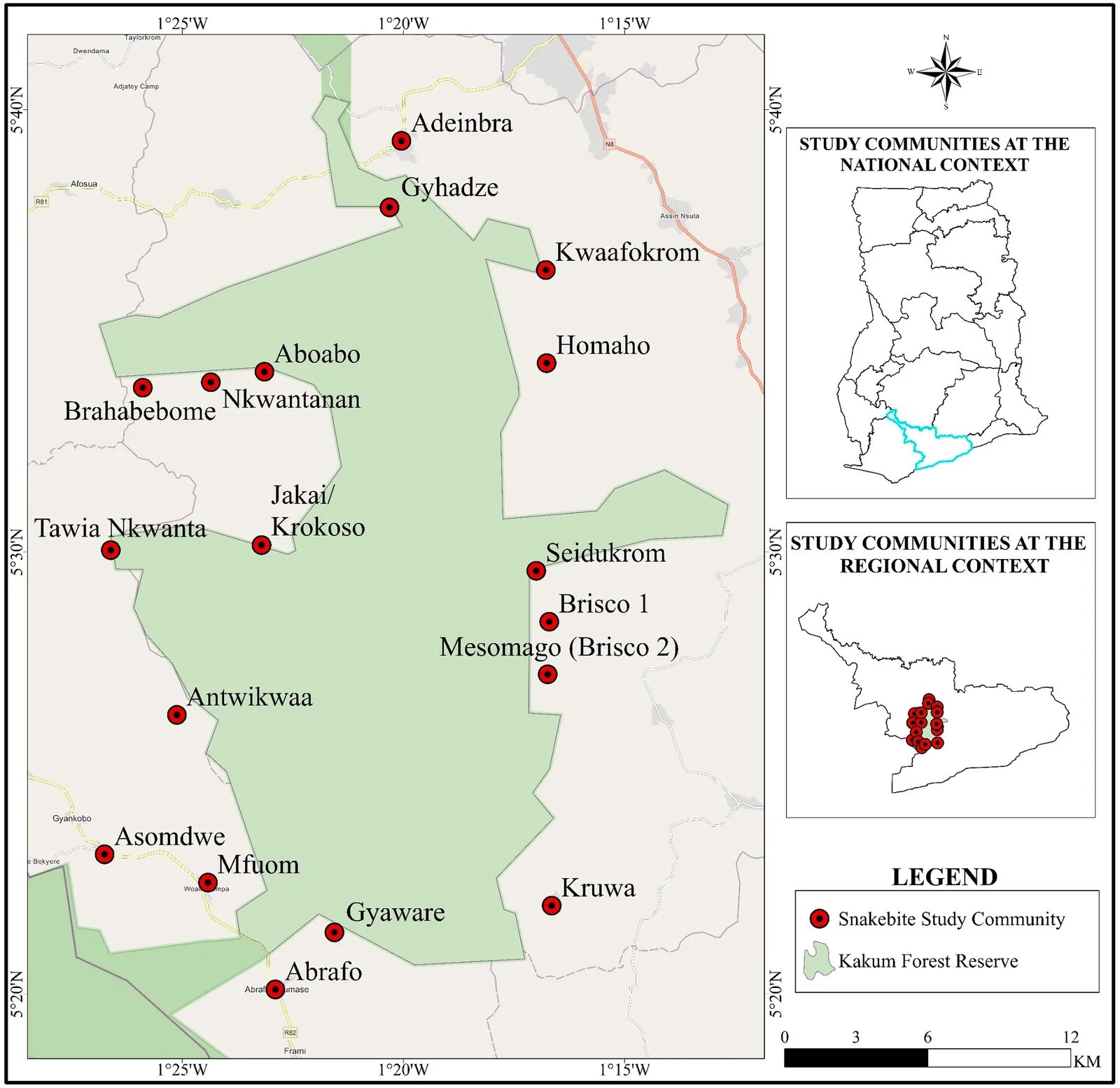
Geo-cardinal points and selected communities in Kakum Conservation Area. Source: Map drawn from GIS shapefiles from Geofabrik’s Ghana extract, which provides OpenStreetMap-derived data. https://download.geofabrik.de/africa/ghana.html, https://download.geofabrik.de/africa/ghana-latestfree.shp.zip, https://www.openstreetmap.org/export.

### Data collection

Data collection for the study took place between April and May 2024, involving two (2) mobile data collection teams each consisting of four (4) enumerators and two (2) supervisors and a local park ranger as a guide and liaison. Prior to the data collection, all team members participated in a two-day training tailored for the purposes of the study. The data collection instrument consisted of six sections: (1) household socio-demographics and environment, (2) snakebite history (lifetime, 5-year, 1-year), (3) snake characteristics (colour, length, setting), (4) symptoms and first aid applied, (5) treatment-seeking behavior (facility type, transport, wait times, alternative care), and (6) outcomes (recovery, disability, death). The data collection instrument had a section for interviewing the head of household and another section for snakebite victims linked with their household related information which could only be activated once a snakebite victim was identified within a household. The data collection instrument was pre-tested involving 30 participants in a selected community (Frami) not included in the main study. Some questions were improved for clarity based on feedback from the pre-testing including questions on snake colour and length which were clarified with visual aids, and the response options for first aid practices were expanded. Cronbach’s alpha for the main scales ranged from 0.72 to 0.86, indicating acceptable internal consistency. The data collection involved the door-to-door enumeration of household heads and snakebite victims using electronic tablets with questions programmed using the KoBoCollect software [23,24]. Interviews were conducted in either English Language or the local language (Fante/Twi), depending on the preference of the respondent. All enumerators were proficient in both English and Fante/Twi languages.

### Data analysis

The data cleaning and analysis were conducted using STATA 17.0 (StataCorp LLC). Both descriptive and inferential statistical techniques were employed in the data analysis. Descriptive statistics included frequencies, proportions, means, and standard deviations with 95% confidence intervals calculated using binomial approximation. Inferential statistics included chi-square tests for bivariate associations and multivariable logistic regression to calculate adjusted odds ratios (AOR) with 95% confidence intervals. Model assumptions (e.g., multicollinearity) were checked using variance inflation factors (VIF < 10, indicating no significant multicollinearity). Statistical significance was set at p < 0.05.

## Results

### Snakebite prevalence and outcomes

Within the period of one year, 41 snakebite cases were recorded among a population of 8,518, representing a prevalence of 0.48% (95% CI: 0.33–0.63) or 4.81 per 1,000 population (95% CI: 3.34–6.29). Over a five-year period, the number of reported cases increased to 164, corresponding to a prevalence of 1.93% (95% CI: 1.63–2.22) or 19.25 per 1,000 population (95% CI: 16.33–22.17). Lifetime exposure to snakebite was highest, with 394 cases reported, yielding a prevalence of 4.63% (95% CI: 4.18–5.07) or 46.26 per 1,000 population (95% CI: 41.80–50.71). Overall, the increasing prevalence rates across longer time horizons indicate a substantial and cumulative burden of snakebite in the KCA population (Table 1 and S1 Fig).

**Table 1:** Snakebite prevalence at the Kakum Conservation Area during the study period.

Period	Snakebite case	Total Population	% (95% CI)	Rate per 1,000 (95% CI)
1-year	41	8,518	0.48 [0.33–0.63]	4.81 [3.34–6.29]
5-year	164	8,518	1.93 [1.63–2.22]	19.25 [16.33–22.17]
Lifetime	394	8,518	4.63 [4.18–5.07]	46.26 [41.80–50.71]

In terms of community specific prevalence of snakebites (Fig 2), there were no reports of snakebite in Gyaware in the last 5 years preceding the survey. However, Brisco-1 and Jakai/Krokoso reported a similarly higher annual prevalence of snakebites (2.26%); while Gyahadze had an even higher (4.63%) 5-year prevalence of snakebites compared with the other communities fringing KCA. In total, six (6) deaths from snakebites were reported, with 3 in the 5-year period before the study and one (1) in the last year prior to the study.

**Fig 2:**
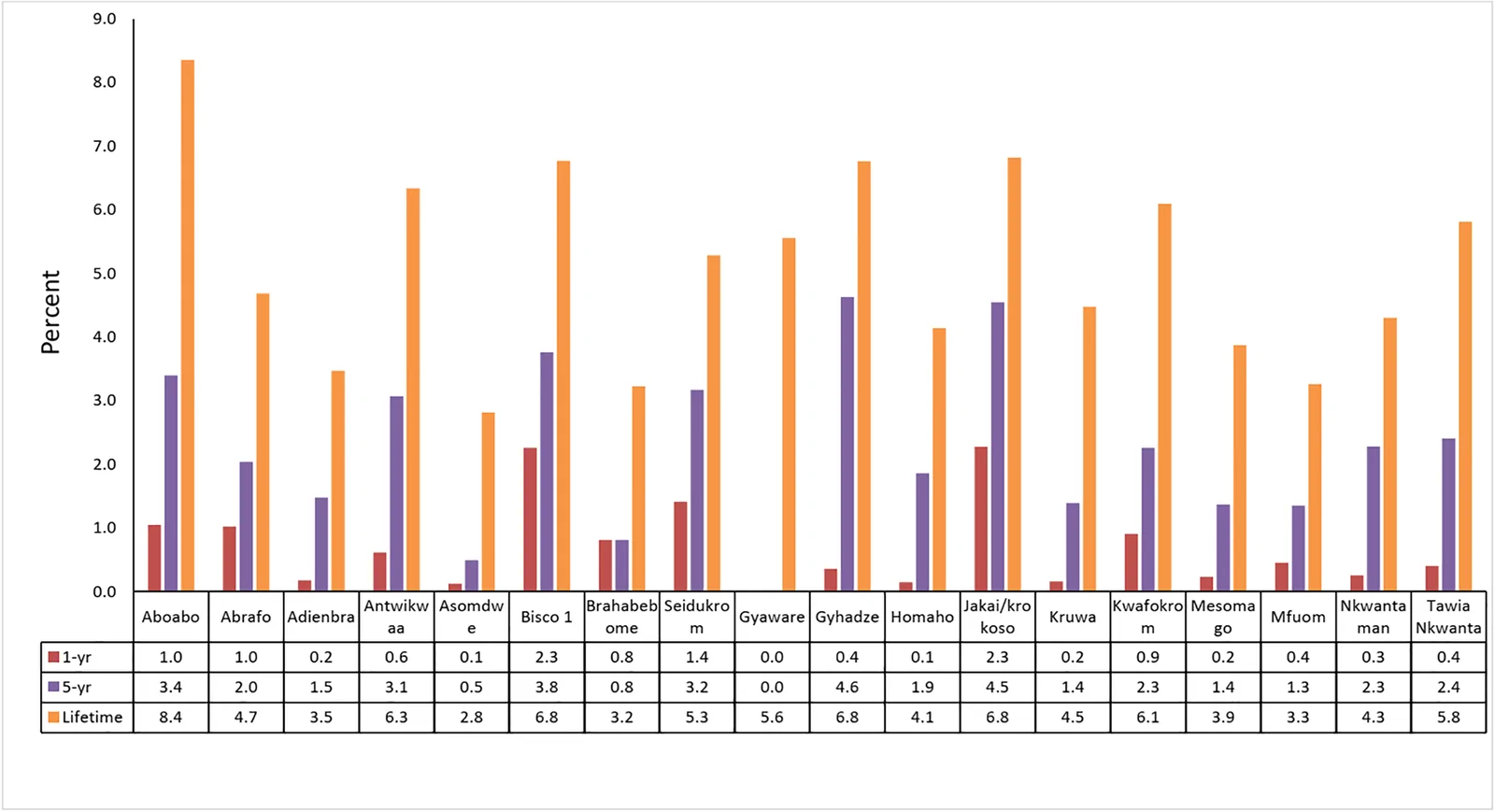
Community specific prevalence of snakebites in KCA.

### Characteristics of snakebite victims, snake features and health seeking behaviors

There were 394 reported cases of snake bites (Table 2). Most victims were aged 40 years and above (χ = 44.69 ± 18.64 years), with the highest proportions in the 40–49 years (23.10%) and ≥60 years (22.34%) age groups, while individuals under 20 years accounted for 10.15%. Females constituted a slightly higher proportion of victims (53.81%) than males (46.19%). Nearly half of the victims had basic education at the junior high school (JHS)/junior secondary school (JSS)/Middle level (47.21%), and 21.32% had no formal education. A majority were married (58.63%) and predominantly engaged in farming (73.35%). The colour of snakes involved was commonly brown (26.90%) or multi-coloured (18.78%), although nearly one-third of victims could not identify the predatory snake. Most bites were from snakes less than 1m in length (43.91%) or of unknown length (24.37%). The lower limbs were the most affected body part (87.56%), and bites occurred mainly in the morning (42.89%) and afternoon (40.10%), largely on farms or in forests (65.48%). The most common symptoms were site pain with swelling (45.69%) or bleeding-related manifestations (29.19%). While most victims applied some form of first aid (74.87%), these were largely inappropriate measures such as tourniquets (34.92%) and herbal applications (25.42%). About 60.66% sought treatment, mainly at Community-based Health Planning Service (CHPS) facilities (32.22%) or hospitals (23.85%), often travelling on foot (40.59%) or by motor vehicle (51.88%), with delays of more than one hour before seeking care reported by many victims. Alternative care was common both before and after facility-based treatment. Despite these challenges, the majority of victims recovered fully (94.67%), although some experienced disability (3.81%) and a small proportion died (1.52%).

**Table 2:** Characteristics of snakebite victims, snake features and related factors at the Kakum Conservation Area.

Characteristic	N	%
**Age group** (Mean = 44.69, ± 18.64)		
<20	40	10.15
20-29	42	10.66
30-39	60	15.23
40-49	91	23.10
50-59	73	18.53
60+	88	22.34
**Sex**		
Female	212	53.81
Male	182	46.19
**Level of education**		
None	84	21.32
Primary	75	19.04
JHS/JSS/Mid	186	47.21
Secondary+	49	12.44
**Marital status**		
Never married	93	23.60
Married	231	58.63
Widow/divorced/separated	70	17.77
**Occupation**		
Non-Farming^†^	105	26.65
Farming	289	73.35
**Colour of the snake**		
Black	39	9.90
Brown	106	26.90
Green	29	7.36
Others	26	6.60
Multi-coloured	74	18.78
Unknown	120	30.46
**Length of the snake**		
<1m	173	43.91
1-2 m	80	20.30
≥3m	45	11.42
Unknown	96	24.37
**Part of body bitten**		
Upper limb	49	12.44
Lower limb	345	87.56
**Time of bite**		
Morning	169	42.89
Afternoon	158	40.10
Night	67	17.01
**Place of bite**		
Pathway/backyard	136	34.52
Farm/forest	258	65.48
**Symptoms following bite**		
Site pain	24	6.09
Site pain/nausea related	75	19.04
Site pain/bleeding related	115	29.19
Site pain/swelling related	180	45.69
**First aid applied**		
No	99	25.13
Yes	295	74.87
**Form of first aid**		
Black stone	21	7.12
Herbs on wound	75	25.42
Tourniquet	103	34.92
Others	96	32.54
**Sought treatment**		
No	155	39.34
Yes	239	60.66
**Place of treatment**		
CHPS facility††	77	32.22
Health centre	36	15.06
Hospital	57	23.85
Herbalist/other	69	28.87
**Means of transport for treatment**		
Other means	18	7.53
On foot	97	40.59
Motor vehicle	124	51.88
**Wait time before seeking care**		
<1 hour	147	61.51
≥1 hour	92	38.49
**Travel time for care**		
<1 hour	150	62.76
≥1 hour	89	37.24
**Form of care provided**		
Black stone	11	4.60
Herbs on wound	52	21.76
Snake bile	23	9.62
Other	153	64.02
**Facility-based care post alternative care**		
No	67	97.10
Yes	2	2.90
**Alternative care post facility-based**		
No	129	75.88
Yes	41	24.12
**Outcome**		
Dead	6	1.52
Disability	15	3.81
Recovery	373	94.67
		
**Total**	**394**	**100.00**

†: Petty trading, unskilled labourer, artisan, fisherfolk, driver, unemployed. †† CHPS = Community-based Health Planning and Services.

### Occurrence of snakebite in a farm/forest by characteristics of snakebite victims and related factors

Table 3 shows the occurrence of snakebite in a farm/forest by characteristics of snakebite victims and related factors. Overall, about two-thirds of snakebites occurred in farms or forests (65.48%), with variations across the various characteristics of victims and other factors considered in the analysis.

**Table 3:** Proportion of snakebite occurrence in a farm/forest by characteristics of snakebite victims.

Characteristic	Snakebite in farm/forest n (%)	Total sample N	X^2^ (df); p-value
**Age**			
<20	20 (50.00)	40	χ²(5) = 5.83; p = 0.32
20-29	29 (69.05)	42	
30-39	43 (71.67)	60	
40-49	62 (68.13)	91	
50-59	47 (64.38)	73	
60+	57 (64.77)	88	
**Sex**			
Female	138 (65.09)	212	χ²(1) = 0.03; p = 0.86
Male	120 (65.93)	182	
**Level of education**			
None	58 (69.05)	84	χ²(3) = 5.23; p = 0.15
Primary	41 (54.67)	75	
JHS/JSS/Mid	124 (66.67)	186	
Secondary+	35 (71.43)	49	
**Marital status**			
Never married	62 (66.67)	93	χ²(2) = 2.64; p = 0.26
Married	156 (67.53)	231	
Widow/divorced/separated	40 (57.14)	70	
**Occupation**			
Non-Farming†	60 (57.14)	105	χ²(1) = 4.40; p = 0.03
Farming	198 (68.51)	289	
**Colour of snake**			
Black	26 (66.67)	39	χ²(5) = 6.18; p = 0.28
Brown	63 (59.43)	106	
Green	21 (72.41)	29	
Others	16 (61.54)	26	
Multi-coloured	56 (75.68)	74	
Unknown	76 (63.33)	120	
**Length of snake**			
<1m	111 (64.16)	173	χ²(3) = 4.68; p = 0.19
1-2 m	60 (75.00)	80	
≥3m	26 (57.78)	45	
Unknown	61 (63.54)	96	
**Part of body bitten**			
Upper limb	41 (83.67)	49	χ²(1) = 8.19; p < 0.01
Lower limb	217 (62.90)	345	
**Time of bite**			
Morning	131 (77.51)	169	χ²(2) = 63.71; p < 0.01
Afternoon	111 (70.25)	158	
Night	16 (23.88)	67	
**Symptoms after bite**			
Site pain	13 (54.17)	24	χ²(3) = 2.56; p = 0.46
Site pain/nausea related	53 (70.67)	75	
Site pain/bleeding related	77 (66.96)	115	
Site pain/swelling related	115 (63.89)	180	
**First aid applied**			
No	71 (71.72)	99	χ²(1) = 2.27; p = 0.13
Yes	187 (63.39)	295	
**Form of first aid**			
Black stone	14 (66.67)	21	χ²(3) = 1.01; p = 0.79
Herbs on wound	49 (65.33)	75	
Tourniquet	67 (65.05)	103	
Others	57 (59.38)	96	
**Sought treatment**			
No	97 (62.58)	155	χ²(1) = 0.95; p = 0.32
Yes	161 (67.36)	239	
**Place of treatment**			
CHPS facility††	47 (61.04)	77	χ²(3) = 2.83; p = 0.41
Health centre	26 (72.22)	36	
Hospital	42 (73.68)	57	
Herbalist/other	46 (66.67)	69	
**Means of transport for treatment**			
Other means	11 (61.11)	18	χ²(2) = 0.34; p = 0.84
On foot	66 (68.04)	97	
Motor vehicle	84 (67.74)	124	
**Wait time before seeking care**			
<1 hour	93 (63.27)	147	χ²(1) = 2.91; p = 0.08
≥1 hour	68 (73.91)	92	
**Travel time for care**			
<1 hour	95 (63.33)	150	χ²(1) = 2.97; p = 0.08
≥1 hour	66 (74.16)	89	
**Form of care provided**			
Black stone	8 (72.73)	11	χ²(3) = 2.19; p = 0.53
Herbs on wound	33 (63.46)	52	
Snake bile	13 (56.52)	23	
Other	107 (69.93)	153	
**Total**	**258 (65.48)**	**394**	

†: Petty trading, unskilled labourer, artisan, fisherfolk, driver, unemployed. †† CHPS = Community-based Health Planning and Services.

The occurrence of snakebite in a farm or forest significantly (p < 0.05) varied across occupation (p = 0.03) of victims, part of body bitten (p < 0.01) and timing of bite (p < 0.01), with most snakebites in the farm or forest occurring among those involved in farming (68.51%), on the upper limp (83.67%) and in the mornings (77.51%). Although not statistically significant, higher proportions of snakebite in a farm or forest occurred among those aged 30–39 years (71.67%), those with secondary or higher education (71.43%), and those married (67.53%). The proportion of snakebites in a farm or forest was similar from both males (65.93%) and females (65.09%). More than seven out of every ten snakebites in the farm or forest were disproportionately occasioned by multi-coloured (75.68%) snakes, 1–2 metres long snakes (75.00%), with majority of victims reported to have site pain and nausea related (70.67%) symptoms. Most victims of snakebite in farms or forest did not apply any form of first aid (71.72%), while about a third of those who applied first aid resorted to the use of black stone, herbs or tourniquet. More than six-in-ten (67.36%) farm or forest snakebite victims sought treatment, with 73.68% of them seeking care in a hospital. The commonest (68.04%) means of transport for care among victims bitten in farms or forests was on foot or motor vehicle, with more than seven-in-ten victims having to wait or travel for over an hour to seek care.

### Snakebite care seeking in a health facility by characteristics of snakebite victims and related factors

Table 4 presents the proportion of snakebite victims who sought care in a health facility following an episode of snakebite by various factors. With respect to background characteristics, snakebite care sought in a health facility was only significantly higher among victims aged below 20 years (67.50%) and those who were never married (52.69%). In terms of other factors, snakebite care sought in a health facility significantly varied by the colour of snake, with a higher proportion of those bitten by “other” coloured snakes seeking care in a health facility. In addition, snakebite care sought in a health facility was significantly higher among victims who used motor vehicles (98.39%) as a means of transport for care, those who waited longer than an hour (84.78%) before seeking care or travelled more than an hour (87.64%) to seek care. Snakebite care sought in a health facility also significantly varied by the form of care provided.

**Table 4:** Proportion of snakebite victims in KCA who sought care in a health facility by their characteristics.

Characteristic	Snakebite Care in Health Facility n (%)	Total Sample N	X^2^ (df); p-value
**Age**			
<20	27 (67.50)	40	χ²(5) = 16.99; p = 0.01
20-29	15 (35.71)	42	
30-39	29 (48.33)	60	
40-49	41 (45.05)	91	
50-59	31 (42.47)	73	
60+	27 (30.68)	88	
**Sex**			
Female	87 (41.04)	212	χ²(1) = 0.83; p = 0.36
Male	83 (45.60)	182	
**Level of education**			
None	26 (30.95)	84	χ²(3) = 11.34; p = 0.01
Primary	43 (57.33)	75	
JHS/JSS/Mid	79 (42.47)	186	
Secondary+	22 (44.90)	49	
**Marital status**			
Never married	49 (52.69)	93	χ²(2) = 9.54; p < 0.01
Married	101 (43.72)	231	
Widow/divorced/separated	20 (28.57)	70	
**Occupation**			
Non-Farming†	49 (46.67)	105	χ²(1) = 0.72; p = 0.39
Farming	121 (41.87)	289	
**Colour of snake**			
Black	23 (58.97)	39	χ²(5) = 16.97; p = 0.01
Brown	38 (35.85)	106	
Green	10 (34.48)	29	
Others	19 (73.08)	26	
Multi-coloured	31 (41.89)	74	
Unknown	49 (40.83)	120	
**Length of snake**			
<1m	65 (37.57)	173	χ²(3) = 5.13; p = 0.16
1-2 m	39 (48.75)	80	
≥3m	24 (53.33)	45	
Unknown	42 (43.75)	96	
**Part of body bitten**			
Upper limb	19 (38.78)	49	χ²(1) = 0.43; p = 0.50
Lower limb	151 (43.77)	345	
**Time of bite**			
Morning	70 (41.42)	169	χ²(2) = 3.71; p = 0.15
Afternoon	64 (40.51)	158	
Night	36 (53.73)	67	
**Place of bite**			
Pathway/backyard	55 (40.44)	136	χ²(1) = 0.62; p = 0.43
Farm/forest	115 (44.57)	258	
**Symptoms after bite**			
Site pain	6 (25.00)	24	χ²(3) = 12.86; p = 0.01
Site pain/nausea related	45 (60.00)	75	
Site pain/bleeding related	46 (40.00)	115	
Site pain/swelling related	73 (40.56)	180	
**First aid applied**			
No	31 (31.31)	99	χ²(1) = 7.54; p = 0.01
Yes	139 (47.12)	295	
**Form of first aid**			
Black stone	11 (52.38)	21	χ²(3) = 1.84; p = 0.60
Herbs on wound	31 (41.33)	75	
Tourniquet	48 (46.60)	103	
Others	49 (51.04)	96	
**Means of transport for treatment**			
Other means	9 (50.00)	18	χ²(2) = 93.94; p < 0.01
On foot	39 (40.21)	97	
Motor vehicle	122 (98.39)	124	
**Wait time before seeking care**			
<1 hour	92 (62.59)	147	χ²(1) = 13.57; p < 0.01
≥1 hour	78 (84.78)	92	
**Travel time for care**			
<1 hour	92 (61.33)	150	χ²(1) = 18.82; p < 0.01
≥1 hour	78 (87.64)	89	
**Form of care provided**			
Black stone	5 (45.45)	11	χ²(3) = 133.17; p < 0.01
Herbs on wound	5 (9.62)	52	
Snake bile	22 (95.65)	23	
Other	138 (90.20)	153	
			
**Total**	**170 (43.15)**	**394**	

†: Petty trading, unskilled labourer, artisan, fisherfolk, driver, unemployed.

### Factors associated with snakebite occurrence in the farm/forest, snakebite care sought in health facility, and recovery from snakebite in KCA

Snakebite occurrence in farm/forest locations was significantly associated with age, marital status, occupation, and travel time to seek care (Table 5). Compared with individuals aged <20 years, those aged 30–39 years had significantly higher odds of experiencing snakebite in farm/forest settings (AOR = 3.39, 95% CI: 1.07–10.67), while elevated but marginal associations were observed among those aged 20–29, 40–49, and ≥60 years. Never-married individuals were significantly more likely to experience snakebite in farm/forest areas compared with widowed/divorced/separated individuals (AOR = 3.83, 95% CI: 1.43–10.28). Farming occupation was also a significant predictor, with farmers having higher odds of farm/forest snakebite compared to non-farmers (AOR = 1.88, 95% CI: 1.04–3.40).

**Table 5:** Factors associated with snakebite occurrence in the farm/forest, care seeking in health facility and recovery following snakebite in KCA.

Characteristic	Snakebite occurrence in farm/forest		Health facility treatment		Recovery from bite	
	AOR (95% CI)	P-value	AOR (95% CI)	P-value	AOR (95% CI)	P-value
**Age**						
<20	Ref.	-	Ref.	-	Ref.	-
20-29	2.35 (0.85, 6.45)	0.10	0.20 (0.05, 0.88)	0.03	7.34 × 10^−4^ (1.12 × 10^−6^, 0.48)	0.03
30-39	3.39 (1.07, 10.67)	0.04	0.36 (0.07, 1.97)	0.24	0.62 (0.02, 15.34)	0.77
40-49	2.81 (0.93, 8.50)	0.07	0.28 (0.05, 1.54)	0.14	0.23 (2.91 × 10^−3^, 18.09)	0.51
50-59	2.45 (0.81, 7.47)	0.11	0.35 (0.06, 2.18)	0.26	0.70 (0.01, 48.03)	0.87
60+	2.98 (0.94, 9.43)	0.06	0.11 (0.02, 0.69)	0.02	0.12 (1.13 × 10^−3^, 12.73)	0.37
**Sex**						
Female	Ref.	-	Ref.	-	Ref.	-
Male	0.91 (0.58, 1.42)	0.67	0.97 (0.42, 2.20)	0.94	1.09 (0.14, 8.62)	0.94
**Level of Education**						
None	Ref.	-	Ref.	-	Ref.	-
Primary	0.56 (0.28, 1.11)	0.10	2.52 (0.68, 9.28)	0.17	0.19 (9.99 × 10^−4^, 35.11)	0.53
JHS/JSS/Mid	0.75 (0.41, 1.37)	0.35	0.83 (0.32, 2.19)	0.71	0.21 (2.10 × 10^−3^, 21.64)	0.51
Secondary+	1.04 (0.42, 2.57)	0.93	2.21 (0.56, 8.71)	0.26	1.00 (1.00, 1.00)	-
**Marital status**						
Widow/divorced/separated	Ref.	-	Ref.	-	Ref.	-
Never married	3.83 (1.43, 10.28)	0.01	2.04 (0.48, 8.60)	0.33	3.09 (0.10, 94.27)	0.52
Married	1.73 (0.91, 3.26)	0.09	2.66 (0.82, 8.59)	0.10	2.69 (0.17, 41.58)	0.48
**Occupation**						
Non-Farming^†^	Ref.	-	Ref.	-	Ref.	-
Farming	1.88 (1.04, 3.40)	0.04	1.56 (0.62, 3.92)	0.35	1.64 × 10^−3^ (2.90 × 10^−6^, 0.93)	0.05
**Colour of the snake**						
Black			Ref.	-	Ref.	-
Brown			0.47 (0.09, 2.47)	0.37	0.69 (4.73 × 10^−3^, 100.00)	0.88
Green			0.47 (0.09, 2.46)	0.37	1.00 (1.00, 1.00)	-
Others			4.83 (0.50, 46.28)	0.17	0.14 (4.05 × 10^−3^, 4.67)	0.27
Multi-coloured			0.36 (0.07, 1.89)	0.23	0.08 (3.22 × 10^−3^, 2.03)	0.13
Unknown			0.23 (0.04, 1.27)	0.09	0.03 (3.52 × 10^−4^, 1.82)	0.09
**Length of the snake**						
<1m			Ref.	-	Ref.	-
1-2 m			1.46 (0.45, 4.72)	0.53	0.54 (0.04, 7.86)	0.65
≥3m			3.07 (0.83, 11.34)	0.09	0.03 (1.82 × 10^−3^, 0.38)	0.01
Unknown			2.60 (0.61, 11.08)	0.20	0.60 (0.02, 21.05)	0.78
**Part of body bitten**						
Upper limb			Ref.	-	Ref.	-
Lower limb			1.09 (0.26, 4.49)	0.91	5.32 (0.20, 141.80)	0.32
**Time of bite**						
Morning			Ref.	-	Ref.	-
Afternoon			0.86 (0.40, 1.87)	0.70	0.36 (0.03, 3.93)	0.40
Night			1.62 (0.50, 5.24)	0.42	0.22 (0.02, 2.32)	0.21
**Place of bite**						
Pathway/backyard						
Farm/forest			1.06 (0.48, 2.36)	0.88	1.13 (0.21, 6.07)	0.89
**Symptoms following bite**						
Site pain			Ref.	-	Ref.	-
Site pain/nausea related			0.98 (0.08, 12.10)	0.99	1.08 × 10^−7^ (5.18 × 10^−9^, 2.25 × 10^−6^)	<0.01
Site pain/bleeding related			0.96 (0.08, 10.97)	0.97	9.57 × 10^−8^ (4.94 × 10^−9^, 1.85 × 10^−6^)	<0.01
Site pain/swelling related			0.34 (0.03, 3.53)	0.36	4.55 × 10^−7^ (1.62 × 10^−8^, 1.28 × 10^−5^)	<0.01
**First aid applied**						
No			Ref.	-	Ref.	-
Yes			1.39 (0.62, 3.13)	0.42	0.35 (0.09, 1.41)	0.14
**Wait time to seek care**						
<1 hour			1.00 (1.00, 1.00)	-	1.00 (1.00, 1.00)	-
_≥ 1 hour			1.43 (0.60, 3.45)	0.42	1.07 (0.17, 6.85)	0.94
**Travel time to seek care**						
<1 hour			Ref.	-	Ref.	-
≥ 1 hour			4.88 (1.98, 12.01)	<0.01	0.38 (0.05, 3.04)	0.36

AOR=adjusted odds ratio; CI=confidence interval; Ref.=Reference; - =not applicable/not estimable.

With respect to care sought in a health facility, younger age groups (20–29 years) and older adults (≥60 years) were significantly less likely to have sought facility-based care compared with those aged <20 years. For recovery outcomes, several factors showed strong associations: individuals aged 20–29 years had significantly lower odds of recovery, and farming occupation was associated with reduced likelihood of recovery (AOR = 1.64 × 10 ^−3^, 95% CI: 2.90 10 ^−6^, 0.93). Snake characteristics and clinical presentation were also important, as longer snake length (≥3 m) and specific symptom profiles, particularly site pain with bleeding or swelling, were significantly associated with recovery outcomes. Overall, the findings highlight that socio-demographic factors, occupational exposure, access-related delays, and clinical manifestations play critical roles in determining where snakebites occur, whether victims seek formal healthcare, and their subsequent recovery in KCA.

## Discussion

This study investigated the epidemiology of snakebite in the fringe communities of the KCA in the Central Region of Ghana. We specifically estimated the prevalence of snakebite, characterized snakebite victims, snake features, health seeking behaviours, and examined associated factors. We found a high prevalence of snakebites in the rural communities fringing the KCA, which is consistent with existing literature indicating that rural dwellers, particularly those living close to forest reserves and wildlife parks, are more exposed to interactions with snake [25,26].The proximity of the KCA to these fringe communities likely increases the risk due to frequent encroachment into natural habitats for farming, fuel wood collection, and other livelihood activities. Other studies in sub-Saharan Africa and rural Asia affirm this high prevalence, noting that poor housing conditions, low awareness, and limited protective clothing contribute to increased exposure [27]. However, some recent interventions, such as awareness campaigns and habitat fencing in other parts of Ghana, have been shown to reduce incidence of snakebite [11], suggesting that targeted preventive efforts could be effective in KCA as well.

The study identified agricultural workers as the most affected group with farmers nearly twice as much to suffer snakebites than non-farmers, reaffirming findings by Sharma et al. [28] and Arias-Rodríguez and Gutiérrez [29], who emphasized the vulnerability of farmers due to their frequent interactions with snake-prone environments. Victims aged 30–39 were more than thrice likely to suffer snakebite on the farm relative to age group <20. Farming activities, especially those involving weeding and harvesting, inadvertently increase the chances of stepping on or disturbing snakes [29]. This finding aligns with a recent Ghanaian study in the Northern Region, where over 70% of snakebite victims were crop farmers [11,16,18]. These findings call for occupation-specific protective measures, including the distribution of boots, gloves, and community training on identifying high-risk zones during peak farming seasons.

The study revealed a relative preponderance of brown-coloured snakes (26.90%) and snakes with a length < 1m (43.91%). These morphological features observed in this study may be suggestive of snakes from the viperidae family. Most viperidae are terrestrial and likely to strike the leg-foot-knee during an attack or bite (87.56%). They are likely to lay in wait by root/stem tubers for rodent pray and may suggest why most bites occurred on the farm and forest areas. The study also revealed site pain/bleeding (29.19%) and site pain/swelling (45.9%) as common occurring symptoms, and these are consistent with haemorrhagic and cytotoxic toxins which is an expressed venom of the viperidae [30]. Site pain/ bleeding was significantly associated with an increased odds of recovery from a bite while site pain/ swelling was the inverse. Overall, symptom severity was significantly associated with recovery, with bleeding- and swelling-related symptoms showing strong associations with recovery outcomes. Meanwhile, snake colours green and black and lengths > 3m were also identified suggesting bites from elapids. However, victims of these bites or their proxies did not recall any neurological symptoms which could be reminiscent of dry bites. This finding is reflected in and validated by further inferential analysis where longer snakes (≥3 m) were associated with reduced odds of recovery. The recognition of multicoloured-blotched snakes adds another layer to the variety of snakes thus providing compelling reason for clinicians to review victims for clinical features before choosing antivenoms for treatment [30].

Most snakebites occurred during the morning and afternoon hours. This temporal pattern corresponds with farming hours, confirming that exposure increases with fieldwork activities. Studies by Warrell [31] and Iliyasu et al. [32] similarly found peak snakebite times to coincide with early and mid-day farming sessions. However, a contrasting study in Nepal reported evening and night bites as most common due to indoor incidents or bites while sleeping [33]. The variation likely stems from different snake species and community habits consistent with the mixed snake typology and varying occupational types expressed in this study but with skewness towards diurnal habits of the snakes and predominantly farming as an occupation. In KCA, where the population is active in the bush during daylight, this daytime pattern makes logical sense. This suggests the need for public education efforts to incorporate time-sensitive cautionary measures and training, particularly targeting the early and mid-day field periods.

The study also highlights the importance of the CHPS facilities as the primary access points in the continuum for care for snakebite envenomation (32.22%). Snakebite victims within the age groups 20–29 and 60+ showed reduced odds of health facility treatment relative to victims age < 20. The CHPS concept reflects Ghana’s policy on decentralizing primary healthcare services and is consistent with findings from Sumah et al. [34] that CHPS facilities play a pivotal role in rural emergency response. Although our findings suggest that CHPS facilities are the preferred point of care for snakebites, perhaps due to proximity and perceived accessibility, we are unable to ascertain from this study the specific form of treatment victims received. However, over a third (35.98%) of snakebite victims in this study received some form of non-orthodox treatment including herbs on wound (21.76%), black stone (4.60) and snake bile (9.62%). This could plausibly reflect the lack of anti-venom and skilled personnel in the CHPS facilities visited, as prior studies [35,36] have noted. Perhaps this is one area worth exploring further in subsequent studies. This may imply that even with limited resources, CHPS facilities are trusted entry points into the health system. Thus, equipping CHPS facilities with snakebite detection kits and related supplies, as well as strengthening referral systems could significantly improve outcomes and reduce complications or fatalities from snakebites in these communities.

The study observed most victims (>60%) sought medical care in less than 1 hour, which diverges from findings in other rural African settings where victims often resorted to traditional healers before visiting formal health centers [37–39]. We also observed that, the victims of snakebite in the KCA with travel time ≥ 1 hour for care had higher odds of seeking care in a health facility; nearly 5-fold more than those with travel time <1 hour. This later finding is reflective in less than 40% of the population of snakebite victims. The observed trend in health seeking behavior of snakebite victims may be attributable to geographical accessibility of health facilities as in the CHPS facilities where most of the victims sought care. This finding contrasts with a study in rural Nigeria where up to 66% of victims first sought herbal treatment [40]. However, in a study in Northern Ghana, similar improvements in health-seeking behavior were recorded in communities that had sustained public health campaigns on snakebite [41], supporting the idea that awareness and trust in the formal health system are growing. This is a promising development which could be leveraged further by integrating community volunteers and local champions to serve as first responders or referral agents during emergencies.

This study provides valuable insight into snakebite trends and health-seeking behaviors in fringe communities of the Kakum Conservation Area. The key strengths of this study include the use of community-level data to situate our findings to local contexts, and its relevance for primary health care planning and delivery. The findings are also timely and useful for informing public health strategies aimed at reducing snakebite-related morbidity and mortality.

However, the study is limited by design structure. Its cross-sectional design limits our ability to establish causal relationships between exposure factors such as occupational activity, time to care etc and health outcomes. Observed associations should therefore be interpreted as correlational rather than causal. In addition, we relied on household heads for the identification of snakebite victims and also on participants’ recollection of snakebite events. Again, we were unable to conduct independent clinical verification of snakebite cases due to the lack of records. These introduce the potential for misclassification bias and recall bias. This may have resulted in under-reporting or misclassification of symptoms, first-aid practices, and care-seeking decisions. For this reason, the prevalence estimates and behavioral patterns may be conservative. Moreover, while the KCA fringe communities share sociodemographic and ecological characteristics with other forest-edge settlements in Ghana, contextual differences in cultural beliefs, health-system access, and environmental conditions may limit the generalizability of the findings to all forest-fringe populations nationwide.

The findings of this study have several key implications for health policy in Ghana. First, there is a critical need to strengthen CHPS facilities particularly in the study area by equipping them with essential snakebite management resources, such as anti-venom, snakebite kits, and trained personnel, particularly in high-risk rural areas. Policies should also integrate snakebite prevention and treatment into national health and occupational safety programmes, especially for agricultural workers. The demonstrated effectiveness of community health education in promoting prompt care-seeking behavior suggests that continuous public sensitization campaigns for snakebites should be supported and expanded.

Our findings highlight the pivotal role of CHPS facilities as the first point of contact for most snakebite victims in rural forest-fringe settings. Given their frontline status, CHPS facilities require enhanced capacity for snakebite management, including the availability of antivenoms, basic resuscitation equipment, and trained personnel capable of early recognition and stabilization of envenomation. Strengthening referral pathways between CHPS facilities and higher-level hospitals is equally essential to reduce delays in the management of severe cases. Another referral pathway could involve educating and empowering traditional healers as agents who link victims to CHPS facilities for care related referrals along the continuum of care.

Moreover, ensuring the distribution of protective gear through agricultural extension services can reduce occupational exposure. Improved data collection and surveillance mechanisms such as incorporating comprehensive snakebite reports into the District Health Information Management System (DHIMS) are necessary for evidence-based planning.

To improve routine monitoring and guide resource allocation, we recommend that snakebite reporting be fully integrated into the District Health Information Management System (DHIMS-2) as part of Ghana’s national NTD surveillance strategy. Incorporating standardized snakebite indicators into DHIMS-2 would enhance national situational awareness and support evidence-based decision-making.

Finally, policy frameworks should promote collaboration between the health sector, forestry and wildlife authorities, agricultural sector and local governance structures to develop coordinated strategies for snakebite prevention, habitat management, and emergency response.

## Conclusion

Snakebites constitute a major public health concern in communities fringing forested conservation areas, especially for people whose livelihood activities revolve around agricultural activities. This study found significant association between farming and snakebite but was unable to establish significant association in clinical outcome (recovery from bite) with timely health seeking behavior and first-aid measures. Most bites occur during morning and afternoon hours when farming activities are at their peak. Overall, the study underscores the need to establish and strengthen collaborative efforts among primary health authorities, forest conservation managers, and community leaders in the design and implementation of effective interventions to avert snakebite incidents and improve outcomes for victims.

## Supporting information

S1 Fig Snakebite prevalence rates per 1,000 population in KCA, with 5% confidence intervals. (TIF)

S1 Table Communities in KCA selected for the study. (DOCX)
