# Supplementary material for: Snakebite epidemiology and health-seeking behavior in fringe communities of the Kakum Conservation Area in Ghana
Source: PLoS Negl Trop Dis. 2026 Oct 30;20(10):e0013288. doi: 10.1371/journal.pntd.0013288 (PMC13739218; doi:10.1371/journal.pntd.0013288)
Supplement: Supplementary file 1 [file pntd.0013288.s002.docx]

S1 Table : Study communities

|  | **Number** | **Percentage, %** |
| --- | --- | --- |
| **North** | **278** | **19.2** |
| Adiembra | 233 | 16.1 |
| Gyahadze | 45 | 3.1 |
| **North East** | **195** | **13.5** |
| Homaho | 117 | 8.1 |
| Kwafokrom | 78 | 5.4 |
| **North West** | **165** | **11.4** |
| Aboabo | 71 | 4.9 |
| Brahabebome | 22 | 1.5 |
| Nkwantaman | 72 | 5.0 |
| **South** | **224** | **15.5** |
| Abrafo | 75 | 5.2 |
| Gyaware | 35 | 2.4 |
| Kruwa | 114 | 7.9 |
| **South East** | **126** | **8.7** |
| Asomdwe | 126 | 8.7 |
| **South West** | **205** | **14.2** |
| Antwikwaa | 80 | 5.5 |
| Mfuom | 125 | 8.7 |
| **West** | **110** | **7.6** |
| Jakai/Krokoso | 28 | 1.9 |
| Tawia Nkwanta | 82 | 5.7 |
| **East** | **142** | **9.8** |
| Brisco 1 | 20 | 1.4 |
| Seidukrom | 44 | 3.0 |
| Mesomago | 78 | 5.4 |
| **Total** | **1445** | **100.0** |
